# Datasets of demographic profile and perpetrator experience in committing crime among young offenders in Malaysia

**DOI:** 10.1016/j.dib.2020.105958

**Published:** 2020-07-02

**Authors:** N.K. Tharshini, Fauziah Ibrahim, Ezarina Zakaria

**Affiliations:** a Universiti Malaysia Sarawak, Malaysia; b Universiti Kebangsaan Malaysia, Malaysia; c Universiti Kebangsaan Malaysia, Malaysia

**Keywords:** Demographic profile, experience, crime, young offenders

## Abstract

The datasets in this article provides supplementary information related to: (1) demographic profile of young offenders and (2) perpetrator experience in committing a crime. A quantitative approach based on a cross-sectional survey design was employed to collect data among 306 young offenders undergoing Community Service Order initiated by the Malaysian Social Welfare Department. The resultant data were analysed descriptively using Statistical Package for the Social Sciences (SPSS). The result stipulates that the majority of respondents are consist of male young offenders aged 20 years old, Malays, single in marital status, and unemployed. Based on the crime involvement aspect, the result indicates that young offenders involved in stealing (26.1%), does not carry any weapons while committing a crime (50.0%), and entangled in criminal activity due to peer influence (40.0%). Moreover, unfavorable luck contributes to the failure in executing crime (52.6%) which subsequently leads them to be arrested by the police (52.0%).

Specifications table

**Table:** 

Subject	Social Sciences
Specific subject area	Social Work and Crime
Type of data	Demographic profile and perpetrator experience in committing crime among young offenders
How data were acquired	Survey among young offenders undergoing Community Service Order
Data format	Raw and analysed (in Excel Worksheet – supplementary file)
Parameters for data collection	Young offenders aged between 18-21 years old
Description of data collection	A quantitative approach was employed to collect data among 306 young offenders undergoing Community Service Order
Data source location	Institution: Malaysia Social Welfare Department City/Town/Region: Kedah, Pulau Pinang, Selangor, Federal Territory of Kuala Lumpur, Melaka, Johor, Kelantan, Pahang Country: Malaysia
Data accessibility	Data is hosted with the article

## Value of the data

• The data can serve as an indication to the Malaysian Social Welfare Department to understand the crime pattern among young offenders in Malaysia.
• The data is valuable to improvise the existing prevention program thus the crime rate among the younger generation can be reduced in the near future.
• The data can be useful for the stakeholders and policymakers working in the fields of crime and social welfare by imposing proper measures to reduce the crime rate among the younger generation in Malaysia.

## Data

The dataset in this article is obtained through a survey conducted among 306 young offenders undergoing Community Service Order. The dataset is divided into two Tables. Table 1 stipules the demographic profile of young offenders whereas Table 2 depicts the perpetrator experience in committing a crime. The raw data file is included as supplementary material in this article.

**Table 1:** Demographic profile of young offenders

Variable (s)	Frequency	Percentage (%)
*Age* 18 years 19 years 20 years 21 years	50 83 111 62	16.3 27.1 36.3 20.3
*Ethnic Group* Malay Indian Chinese	277 15 14	90.5 4.9 4.6
*Marital Status* Single Married	289 17	94.4 5.6
*Occupation* Student Unemployed Full-Timer Part-Timer	43 152 51 60	14.1 49.7 16.7 19.5

**Table 2:** Perpetrator experience in committing a crime

Variable (s)	Frequency	Percentage (%)
*Types of Crime* Stealing Traffic Burglary Drugs Snatch Thief People-Related Weapon/Fire Arm Gamble Infringement of Supervision Terms	80 71 55 35 31 16 8 8 2	26.1 23.2 18.0 11.7 10.1 5.2 2.6 2.6 0.5
*Usage of Weapon* No weapon was used Steel Rod Machete Knife Duplicate Key Knife Screw Driver Spanner Wire Cutter	153 57 36 19 16 13 9 3	50.0 19.0 11.4 6.2 5.2 4.2 3.0 1.0
*Factors Associated to Commit a Crime* Peer Influence Self-Satisfaction Desperate Need of Money Unemployed Buying Drug Paying Debt Revenge Others *Factors Associated to Failure In Committing a Crime* Unfavourable Luck Unable to Escape Tight Police Surveillance *Caught By* Police Public Friend Informed Police Family Member Informed Police Others	122 74 56 27 17 6 3 1 161 107 38 161 86 34 22 3	40.0 24.1 18.3 8.8 5.6 2.0 0.9 0.3 52.6 35.0 12.4 52.0 28.7 11.1 7.2 1.0

## Experimental design, materials, and methods

### Experimental design

A quantitative approach based on a cross-sectional survey design was employed to collect data among 306 young offenders undergoing Community Service Order. Nine survey questions were developed based on previous studies in the field of crime [1,2]. Upon developing the instrument, face validity and content validity were executed to ensure that the developed items in the instrument represent the measured phenomena. In general, face validity refers to the researcher's subjective assessment to verify whether the items in the instrument appear to be relevant, clear and reasonable [3]. Correspondingly, according to Anastasi and Urbina (1997) [4] content validation plays a primary role to test the accuracy of the domain that is aimed to be measured.

Face validity was employed by getting feedback from the subject matter expert (panel) to review and validate all the items (question) within the instrument. Five panels were selected based on their expertise in the field of psychology, crime, community development, social work, and statistical data analysis. Specific guidelines were also used for selecting the experts including; (i) experienced academicians (more than 5 years) and (ii) familiar with evidenced-based practice (teach or publish articles in their field of expertise) [5]. Table 3 shows the expertise and years of experience of the panels.

**Table 3:** Expertise and year of experience of the panels

Panel	Expertise	Experience (Year)
1	Developmental Psychology, High-Risk Behavior	More than 5 years
2	Criminology, Criminal Psychology, Forensic Science	More than 5 years
3	Community Development, Community Education and Human Development	More than 5 years
4	High-Risk Children and Adolescent	More than 5 years
5	Test and Measurement, Statistics, SEM Model Testing	More than 5 years

The criteria for face validity assessment for this study is based on Oluwatayo (2012) [3] guidelines that focus on six main aspects namely; (i) unambiguity items, (ii) appropriate grammar, (iii) correct sentence structure, (iv) correct spelling, (v) proper format and structure of the instrument, and (vi) appropriate font size. Moreover, the panel was also requested to provide additional suggestions and comments to improvise the instrument. The summary of the panel's comments for face validity is shown in Table 4.

**Table 4:** Summary of the panel's comments for face validity

Panel	Comment
2,4	Improvise the sentence structure
1,5	Split the double-barrel questions
1,2,3,4,5	Format acceptable
3	Simplify the language

Amendments to the instrument were done after obtaining feedback from the panels. Following this, content validity was carried out to provide evidence about the degree to which the developed instrument is relevant to the targeted construct. The content validity of the instrument was established based on the Content Validity Index (CVI) where an item is considered not relevant if the CVI score is less than 0.78 [5]. In addition, a dichotomous rating of favorable or unfavorable was also used to quantify the content validity [6,7]. Favorable denotes that an item is relevant and concise [8]. As a result, these items are assigned a score of +1.0 [7]. On the contrary, unfavorable denotes that an item is irrelevant or negligible [8]. Hence, these items were given a score of +0.00 [7].

For this study, a favorable rating by three or more members of the expert panel and a CVI greater than 78% = 0.78 indicates that the items (questions) are considered relevant/related to the topic of study. Table 5 stipulates the content validity index of the study.

**Table 5:** Content Validity Index (CVI)

No.	Variable (Part A – Demographic Profile)	Number in Agreement (Panels)	CVI
1.	Age	5	1.00
2.	Ethnic Group	5	1.00
3.	Marital Status	5	1.00
4.	Occupation	5	1.00
No.	Variable (Part B – Perpetrator Experience In Commiting Crime)	Number in Agreement (Panels)	CVI
1.	What offense did you commit?	5	1.00
2.	What is the weapon that was used while commiting the crime?	5	1.00
3.	What is the main factor that leads you to commit the crime?	5	1.00
4.	What is the main factor/reason that leads to the failure in commiting the crime?	3	0.78
5.	Who caught you?	3	0.78
	Total		8.56
	Propotion favorable		8.56/9 = 0.951

The final survey questions are as below:

**Table:** 

SECTION A: DEMOGRAPHIC PROFILE			

1. Age		: _________	
2. Sex			
1	Male		
2	Female		
3. Ethnic Group			
1	Malay	3	Indian
2	Chinese	4	Others
4. Marital Status			
1	Single	3	Others
2	Married		
5. Occupation			
1	Student	3	Unemployed
2	Full-Timer	4	Part-Timer

SECTION B: PERPETRATOR EXPERIENCE IN COMMITTING A CRIME			

1. What offense did you commit?			
1	Stealing	6	People Related Crime
2	Traffic Related Crime	7	Weapon/Fire Arm
3	Burglary	8	Gambling
4	Drug Abuse	9	Infringement of Supervision Terms
5	Snatch Thief	10	Others
2. What is the weapon that was used while committing the crime?			
1	No Weapon Was Used	5	Screw Driver
2	Steel Rod	6	Spanner
3	Machete Knife	7	Wire Cutter
4	Duplicate Key	8	Others
5	Knife		
3. What is the main factor that leads you to commit the crime?			
1	Peer Influence	5	Buying Drug
2	Self-Satisfaction	6	Paying Debt
3	Desperate Need of Money	7	Revenge
4	Unemployed	8	Others
4. What is the main factor that leads to the failure in committing the crime?			
1	Unfavourable Luck	3	Tight Police Surveillance
2	Unable to Escape	4	Others
5. How do you get caught?			
1	Arrested By The Police	4	Family Member Informed Police
2	Public Informed Police	5	Others
3	Friend Informed Police		

### Research design

A cross-sectional survey design was used to complete the data collection process. According to Malhotra et al. (1996) [9], a cross-sectional survey design is a method that involves data collection from a selected population within a specific time based on the attribution of the current respondent.

### Population

In this study, the population refers to all the young offenders undergoing Community Service Order initiated by the Malaysian Social Welfare Department. A report obtained from the Malaysian Social Welfare Department disclosed that currently, a total number of 540 young offenders are actively undergoing the Community Service Order.

### Sample and location of study

A sample refers to a smaller and manageable version of a larger group. According to Sangoseni et al. (2013) [7], a sample is a subset containing the characteristics of a larger population. The sample size in this study was determined based on Sample Size Calculator developed by Cohen et al. (2001) [10] whilst taking into consideration the significant level at p<.05 (significant level = 95%). Based on Cohen's Sample Size Calculator, if the population of the study is 540 and the level of significance required is .05 thus the number of respondents needed for the study is 278 respondents. Taking into consideration aspects such as dropout rate and errors in filling up the survey by the respondents, the researchers agree to increase the sample size up to 10%. Therefore, the sample size for this study is 306 respondents. Assuredly, Abdul Ghaffar (1999) [11] have supported that enlarging the sample size will help to elevate the reliability and validity scores of a particular study.

Stratified random sampling was used to select the young offenders from four different zones in Malaysia namely; (i) North Zone, (ii) Central Zone, (iii) East Zone, and (iv) Southern Zone. According to Hayes (2020) [12], stratified random sampling allows a researcher to obtain a sample that best represents the entire population that is being studied. In the context of this study, stratified random sampling was employed in order to create equitable representation from the total population since the number of young offenders within each zone was different.

Two institutions with the highest number of young offenders within each zone was selected as the location of study including; North Zone – Kedah and Pulau Pinang, (ii) Central Zone – Selangor and Federal Territory of Kuala Lumpur, (iii) East Zone - Pahang and Kelantan, and (iv) Southern Zone – Melaka and Johor. The cut-off number for an institution to be selected as the location of the study is at least by having a minimum number of 35 young offenders who are actively undergoing Community Service Order. These criteria were included since it is cost-effective to focus on zones with a higher number of young offenders. Table 6 depicts the location of the study.

**Table 6:** Location of the study

Zone	Social Welfare Department (SWD)	Population of Young Offenders
North	SWD in Perlis **SWD in Kedah** **SWD in Pulau Pinang** SWD in Perak	13 **48** **36** 20
Central	SWD in Negeri Sembilan **SWD in Selangor** **SWD in Federal Territory of Kuala Lumpur**	64 (*this population were excluded during the data collection – already used as respondents for pilot study*) **72** **68**
East	**SWD in Pahang** **SWD in Kelantan** SWD in Terengganu	**79** **36** 25
Southern	**SWD in Melaka** **SWD in Johor**	**42** **37**
	TOTAL	540

### Ethical considerations

High values and norms were upheld throughout the data collection process. The participation of the respondent in this study is strictly voluntary. Prior to participation, the researcher's explained to the respondents regarding the purpose of the study. After consent was given, respondents were assured that all their responses will be recorded confidentially and reported anonymously. Moreover, respondents were also informed that they could withdraw at any stage of the study without repercussions. Furthermore, no incentives were provided to encourage participation.

### Procedure

The survey questions were disseminated by the researcher to the respondents after getting permission from Malaysian Department of Social Welfare (JKMM 100/12/2/2:2016/013). During the data collection process, the researcher's assist and clarify all the questions asked by the respondents regarding the survey questions. Moreover, respondents were also informed about their rights to confidentiality. Thus, all the respondents were reminded not to write their names or other personal information on the given materials. There was no time limit for the respondents to answer the survey questions. Approximately, respondents took about 15-20 minutes to complete the questionnaire.

### Data analysis

Descriptive analyses were used to obtain information related to frequency and percentage. Data were analysed using Statistical Package for the Social Sciences (SPSS).

## Declaration of Competing Interest

There is no conflict of interest regarding the research, publication, and authorship of this article.
